# A new minimally invasive technique for the repair of diastasis recti: a pilot study

**DOI:** 10.1007/s00464-021-08393-2

**Published:** 2021-03-04

**Authors:** Gabriele Manetti, Maria Giulia Lolli, Elena Belloni, Giuseppe Nigri

**Affiliations:** 1 Department of General Surgery, St. Giovanni Addolorata Hospital, Rome, Italy; 2 Department of Medical and Surgical Sciences and Translational Medicine, St. Andrea University Hospital, Sapienza University of Rome, Via di Grottarossa 1035/1039, 00189 Rome, Italy

**Keywords:** Diastasis recti, Surgical technique, Abdominal wall reconstruction

## Abstract

**Background:**

Diastasis recti is an abdominal wall defect that occurs frequently in women during pregnancy. Patients with diastasis can experience lower back pain, uro-gynecological symptoms, and discomfort at the level of the defect. Diastasis recti is diagnosed when the inter-rectus distance is > 2 cm. Several techniques, including both minimally invasive and open access surgical treatment, are available. Abdominoplasty with plication of the anterior rectus sheath is the most commonly used, with the major limitation of requiring a wide skin incision. The new technique we propose is a modification of Costa’s technique that combines Rives–Stoppa principles and minimally invasive access using a surgical stapler to plicate the posterior sheaths of the recti abdominis.

**Methods:**

It is a fully laparoscopic technique. The pneumoperitoneum is induced from a sovrapubic trocar, placed using an open access technique. The posterior rectus sheath is dissected from the rectus muscle using a blunt dissector to create a virtual cavity. The posterior sheets of the recti muscles are plicated using an endo-stapler. A mesh is then placed in the retromuscular space on top of the posterior sheet without any fixation. Using a clinical questionnaire, we analyzed the outcomes in 74 patients who underwent minimally invasive repair for diastasis of the rectus abdominis sheath.

**Results:**

Seventy-four patients (9 men and 65 women) were treated using this technique. Follow-up was started two months after surgery. All procedures were conducted successfully. There were no major complications or readmissions. No postoperative infections were reported. There were two recurrences after six months. There was a significant reduction in symptoms.

**Conclusions:**

This new method is feasible and has achieved promising results, even though a longer follow-up is needed to objectively assess this technique.

**Supplementary Information:**

The online version contains supplementary material available at 10.1007/s00464-021-08393-2.

Diastasis recti is a very common acquired condition in which the rectus abdominis muscles are separated by an abnormal distance along their lengths. Unlike hernias, there is no fascial defect. The minimum inter-rectus distance to define a diastasis is 2 cm. The condition is due to an increase in intra-abdominal pressure in which the forces applied to the linea alba cause it to stretch, resulting in a widening of the inter-rectus distance. For these reasons, it occurs most frequently after pregnancy, but obesity or previous abdominal surgeries can also be the cause [1, 2].

Umbilical and epigastric hernias are often associated with diastasis due to the progressive laxity of the midline fascia. Surgery should correct diastasis and hernia at the same time, due to the high risk of recurrence if only the hernia is treated.

Patients with diastasis can experience lower back pain, uro-gynecological symptoms, such as urinary incontinence, and pelvic organ prolapse. Discomfort at any level along the defect may be reported. Moreover, the appearance of the abdominal wall is significantly altered, especially during contraction of the rectus abdominis muscles [2, 3]. Therefore, diastasis recti has esthetic implications.

Several surgical procedures are available for the management of recti diastasis, both open and laparoscopic. The choice of technique depends mainly on the inter-rectus distance and the laxity of the anterior abdominal wall, even if there are no clear guidelines. For mild to moderate recti diastasis, simple plication of the linea alba is usually considered. The plication-based techniques include open plication, laparoscopic plication, or hybrid plication of either the anterior or posterior rectus fascia. Plication can be performed with single- or double-layer sutures, using an interrupted or continuous, absorbable, slowly absorbable, or permanent sutures according to the surgeon’s preferences [2–4]. In the case of coexistence of extensive laxity of the abdominal wall, onlay mesh reinforcement is generally used, even though there is a lack of evidence on which type of mesh should be used.

In case of moderate to severe diastasis, retrorectus repair reinforcement with sublay mesh, based on the Rives–Stoppa principles, should be considered [5, 6]. The placement of a mesh in a retrorectus plane allows greater improvement in muscular strength and provides the most durable repair, with no adherence to bowel loops [7]. Postoperative complications, include infection, seroma, mesh extrusion, recurrence, nerve injury, postoperative pain, skin necrosis, and visceral injury.

The laparoscopic procedure we describe combines the Rives–Stoppa principles with mechanical plication of the rectus fascia using a stapler. Costa et al. described a similar technique for incisional hernia repair in patients who previously underwent laparoscopic gastric bypass [8–10].

We decided to apply a modified Costa’s technique as a treatment option in patients affected by diastasis recti, and we aimed to overcome some concerns raised by this technique when used in ventral hernias repairs, such as hernia width, redundant hernia sac left in the subcutaneous tissue, and the persistence of the pervious surgical scar [9].

The aim of this study was to evaluate the effectiveness and feasibility of using a laparoscopic approach to perform mechanical plication of the rectus fascia with a diastasis recti > 2 cm using a linear endoscopic stapler.

## Methods

### Patients inclusion

Between April 2019 and July 2020, 74 patients (9 men and 65 women) were treated using this new technique. Informed consent was obtained from all individual participants included in this study. The procedure was in accordance with the ethical standards of the institutional research committee and with the 1964 Helsinki declaration.

Inclusion criteria were as follows: recti abdominis diastasis > 2 cm, symptomatic patients, and body mass index (BMI) between 17 and 35. Exclusion criteria were as follows: recent pregnancy (at least 6 months), oncological patients, and patients who had a previous abdominal wall surgery with the positioning of a mesh. The largest recti diastasis observed was 12 cm.

A polypropylene or composite mesh was used in the retrorectus pocket, and no fixation device was used for the mesh. The initial opening of the posterior sheath of the rectus abdominis muscles was closed with a barbed absorbable suture. Mesh was placed in all patients.

All patients were requested to fill out a preoperative questionnaire about symptoms associated with their diastasis before and at 2 and 6 months after surgery, simultaneously with clinical examination. Urinary incontinence, lower back pain, shortness of breath, and abdominal swelling were evaluated.

### Surgical technique (Video included)

**Fig. 1:**
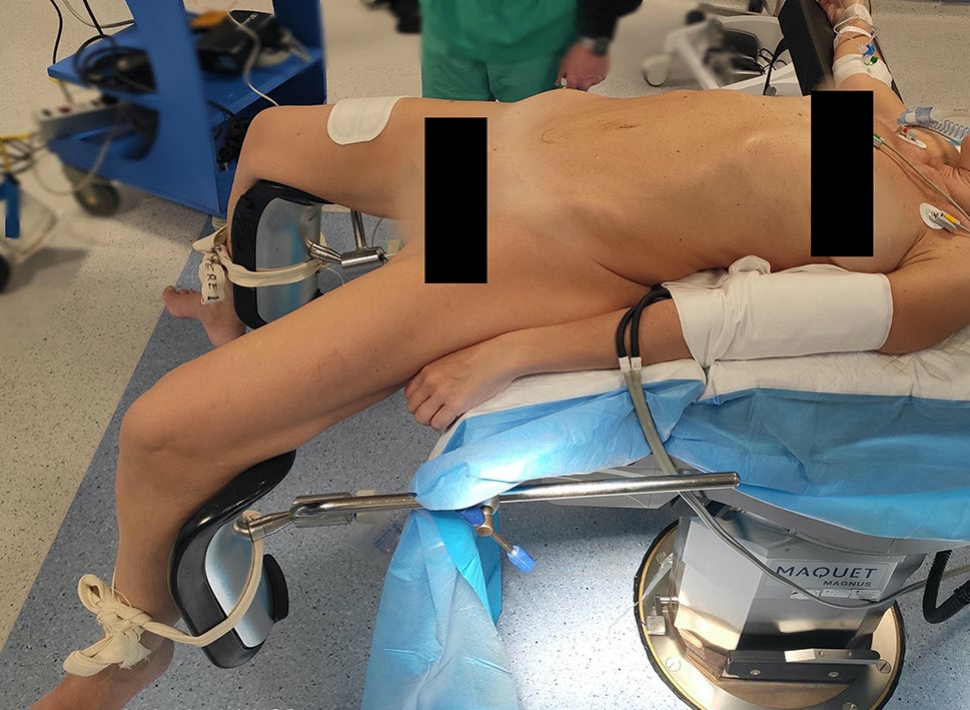
Patient’s position

The patient is placed in a supine/combined lithotomy position: thighs were extended (120°) (Fig. 1). Preoperative antibiotics are administered, and general endotracheal anesthesia is induced. A Foley catheter is placed and then removed at the end of the procedure. The abdomen is prepped and draped in the usual sterile fashion.

Pneumoperitoneum is induced using an open technique, placing the first 12-mm trocar 2 cm above the pubic symphysis. The abdomen is insufflated with carbon dioxide up to a pressure of 12 mmHg.

A 30° laparoscope is inserted, and the abdomen and abdominal wall are inspected.

**Fig. 2:**
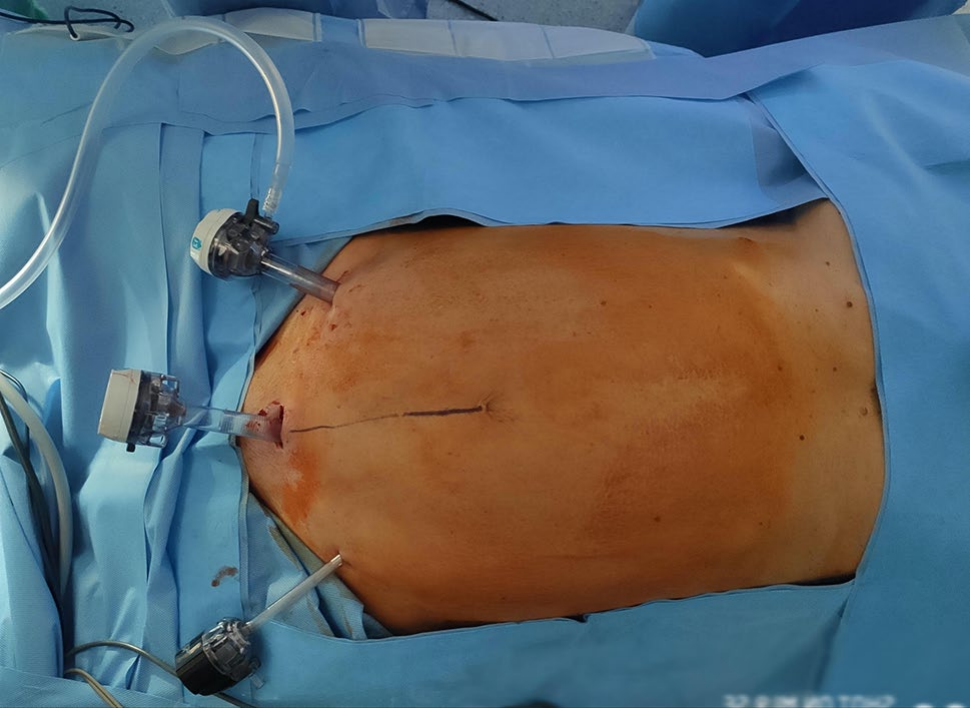
Ports’ position

**Fig. 3:**
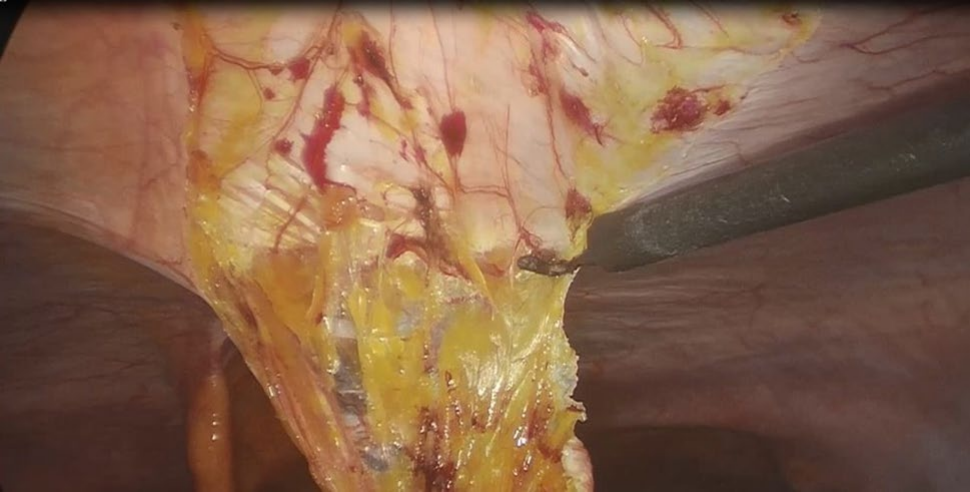
Dissection of the preperitoneal adipose tissue

Additional trocars are inserted under direct vision in the following locations: a 12-mm trocar in the left iliac fossa and a 5-mm trocar in the right iliac fossa. If a 5-mm laparoscope is available, two 5-mm trocars and one 12-mm trocar can be used (Fig. 2). When necessary, lysis of adhesions to the abdominal wall is performed. Hernias possibly encountered along the median line are reduced into the abdominal cavity and the sac is resected. Redundant adipose tissue is dissected from the posterior layer of the anterior abdominal wall until the defect is exposed (Fig. 3), and the falciform ligament is dissected.

**Fig. 4:**
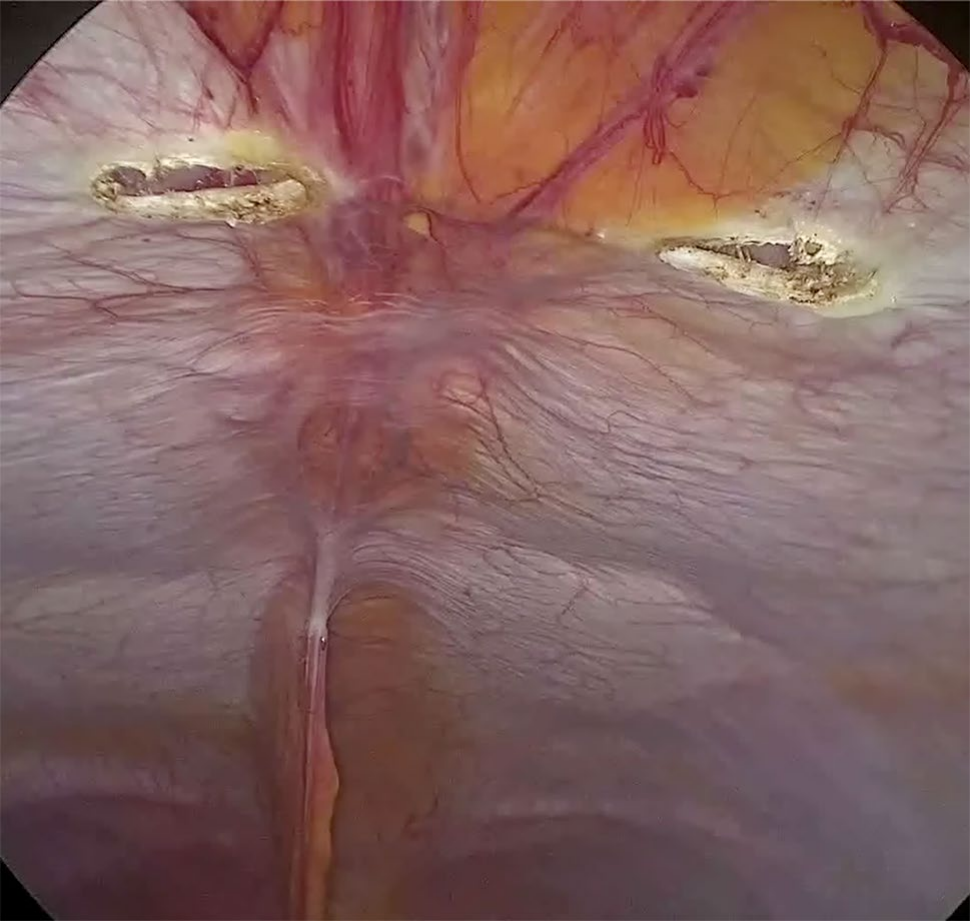
Peritoneum and posterior sheath opening

The peritoneum and the posterior rectus sheaths are incised bilaterally where the diastasis begin, about 3 cm below the umbilicus, using a monopolar energy device. Then, the retromuscular space is exposed (Fig. 4).

**Fig. 5:**
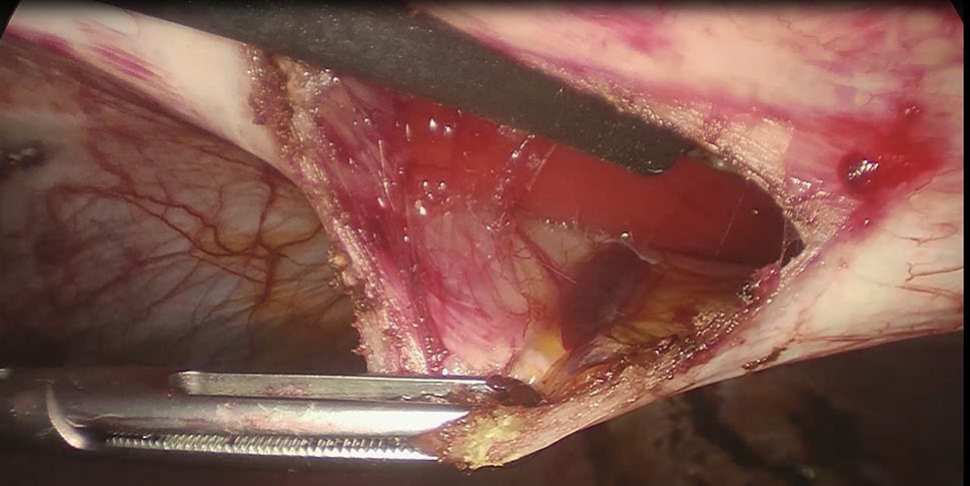
Dissection of the posterior sheath from the muscle

**Fig. 6:**
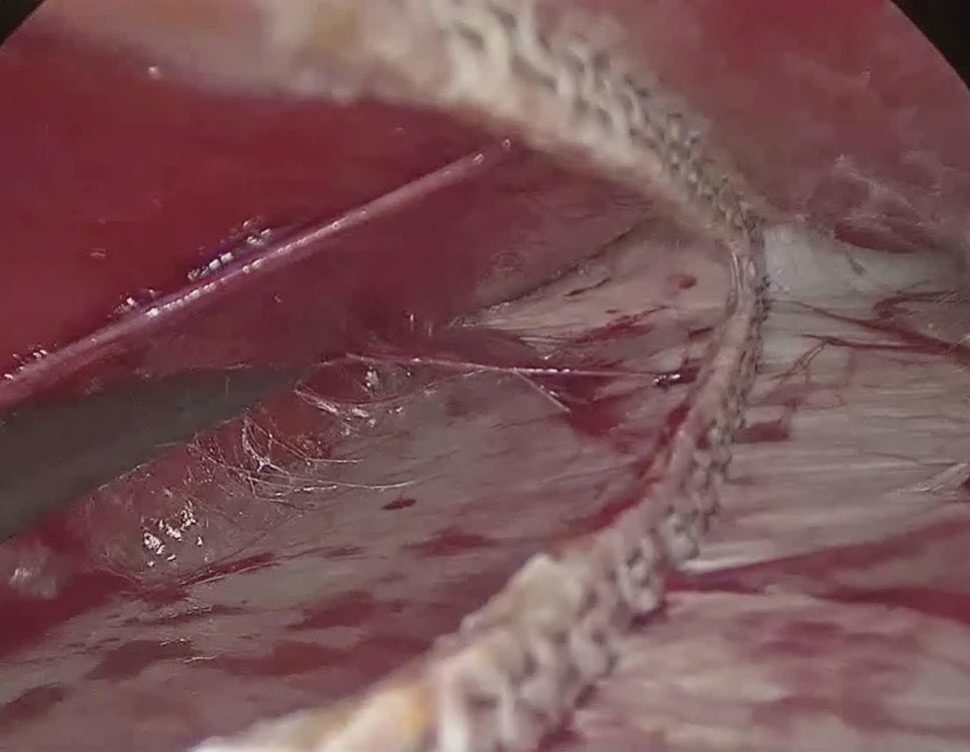
Pocket between muscles and posterior sheath

**Fig. 7:**
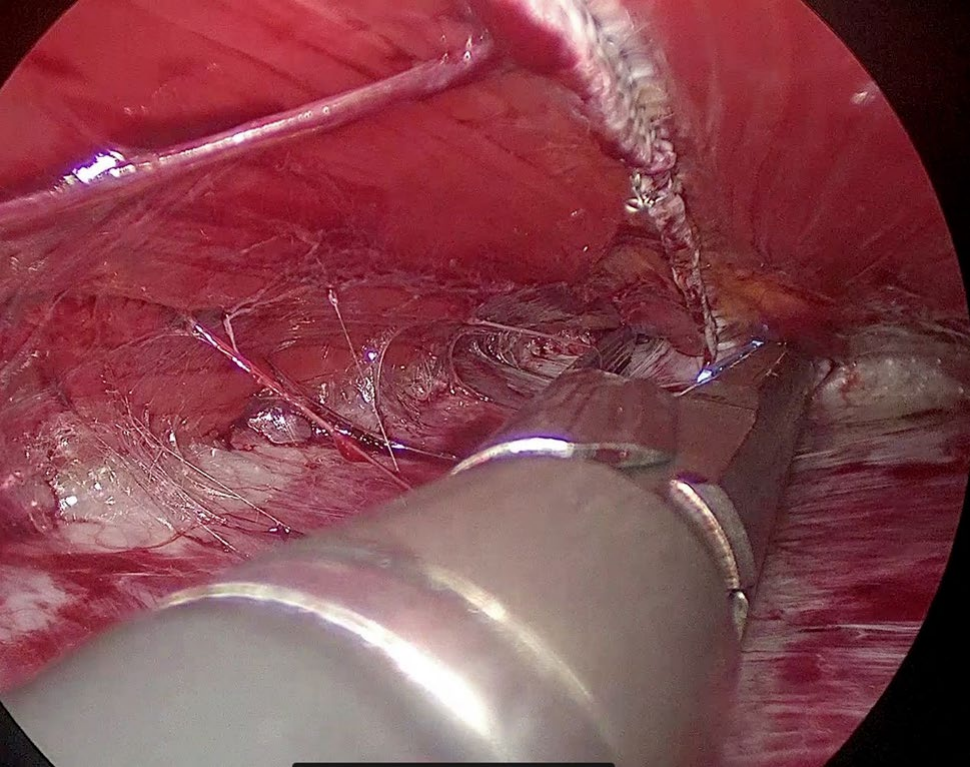
Last stapler fired close to the xiphoid process

**Fig. 8:**
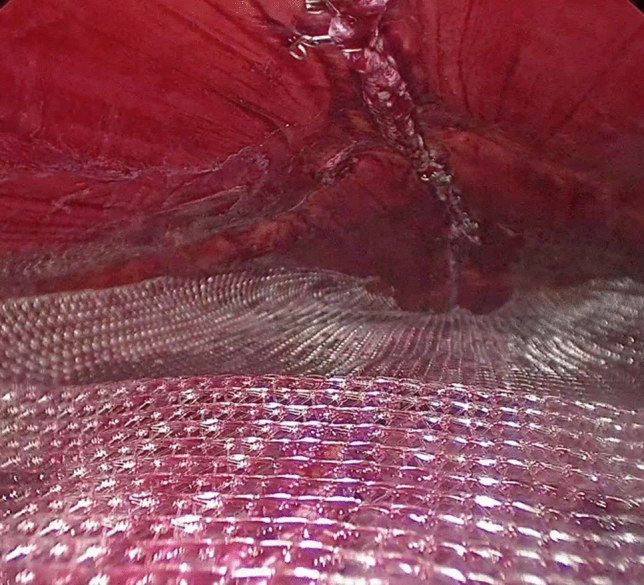
Mesh placement

At this point, the laparoscope is moved to the trocar in the left iliac fossa. The rectus sheath is dissected from the muscle using a blunt dissector introduced in the space previously created (Fig. 5). The abdominal pressure is lowered to 6–7 mmHg. A 60-mm endoscopic stapler (blue load), possibly reinforced, is introduced through the suprapubic trocar. Its jaws are opened and then they are introduced into the two retromuscular pockets previously created, 3 cm above the umbilicus. The stapler is fired, joining together the rectus sheaths. After this procedure, a cavity is created between the rectum muscles anteriorly, and their sheaths posteriorly (Fig. 6). The dissection of the muscle from the sheath is carried out cranially and laterally to reduce lateral tension and to reach the xiphoid process. During this procedure, dissection should reach the edge between the rectus muscle and the transverse muscle laterally, and the lower margin of the ribcage cranially. The firing process is repeated in a cranial direction until the xiphoid process is reached (Fig. 7). A polypropylene or dual mesh is placed in the space between the rectus muscles and the posterior sheaths without using any fixation device (Fig. 8).

Subsequently, the laparoscope is moved back to the suprapubic trocar. A barbered absorbable suture is used to close the initial opening in the posterior sheath. A transverse abdominis plane block is performed using ropivacaine 7.5 mg/mL in 20 mL of saline solution. The trocars are removed under direct vision. Accesses for trocars greater than 5 mm are closed with 2.0 polyglactin suture at the fascial layer. At the end of this procedure, the medial margins of the rectus muscles are approximated at the midline, thereby repairing the diastasis and potential midline hernias. Very large diastases can be treated with Botox infiltration before surgery to ensure a greater laxity of the muscle structures.

## Results

The mean age was 46.3 (SD ± 11.3) years (Table 1). There were 9 men and 65 women. The mean BMI was 24.3 (SD ± 4.1). The mean diastasis width was 4.7 (SD ± 1.5), as measured in the supra-umbilical region. Forty patients were affected by diastasis recti associated with umbilical hernia. In 10 patients, it was associated with epigastric hernia. Abdominoplasty was performed after laparoscopy in 20 patients. Midline ventral hernia was repaired using the same technique in 4 patients. Characteristics of the patient population are shown in Table 1.

**Table 1:** Patients characteristics

Mean age	46.3 (SD 11.3)
Men/women	9/65
BMI	24.3 (SD 4)
Diastasis size	4.7 (SD 1.5)
Associated umbilical hernia	40
Associated epigastric hernia	10

All procedures were performed using the same technique and none needed open conversion.

The average duration of surgery was 90 min. When only recti diastasis was corrected, patients were discharged on postoperative day 2, while hospitalization was 4 days on average if an abdominoplasty was associated. One patient had a subcutaneous hematoma treated conservatively, while another patient presented with postoperative bleeding when abdominoplasty was performed at the same time. No postoperative infections or seromas were reported. There have been no readmissions, and only two recurrences have been reported 6 months after surgery.

On postoperative day one and two, 3 g of paracetamol was administered to all patients and 10 mg of ketorolac was administered to patients with persistent pain after surgery. A survey among patients showed a progressive reduction of postoperative pain until its disappearance on postoperative day 7. The median visual analog scale (VAS) score on the first postoperative day was 5.5 ± 2. The average follow-up period was 6 months (range 2–12 months).

Of all patients, 57 (77%) completed the preoperative and postoperative questionnaires.

Of all the patients, 31 (42%) had urinary incontinence before the surgery, while only 2 patients reported this symptom after surgery. Lower back pain was reported before surgery in 40 patients (54%), and 36 patients (90%) experienced improvement. 21 patients (28%) had respiratory symptoms, mostly shortness of breath, and 18 patients (86%) reported improvement with diastasis correction. Abdominal swelling and feeling of distress were also common symptoms in 43 patients (60%), with a postoperative improvement of 84%. In fact, only 7 patients reported this symptom 6 months after surgery. Even though these data are preliminary and based on patients’ perception and personal experience, they seem promising. Further follow-up studies are needed. The results are shown in Table 2.

**Table 2:** Symptoms assessment before and after surgery: *UI* urinary incontinence, *LBP* lower back pain, *SB* shortness of breath, *AS* abdominal swelling

	UI (%)	LBP (%)	SB (%)	AS (%)
Preoperative symptoms	42	54	28	60
Postoperative symptoms	3	5	4	9

## Discussion

The Rives–Stoppa technique was first described in the mid-1980s and since then, it has played a major role in abdominal wall surgery. To date, even though several other techniques have been described, it is still considered the gold standard.

The sublay position of the mesh proved to have the lowest risk of long-term recurrence and major complications due to the stability of the retromuscular mesh and absence of contact with the abdominal content [11, 12]. According to the La Place principle, a large overlap of underlay mesh allows the distribution of forces over the entire mesh, decreasing the pressure on the fascia defect, leading to reduced recurrence rate [13]. For these reasons, a retromuscular proper-sized mesh is held in place by intra-abdominal pressure, making fixation unnecessary, and thus reducing postoperative pain, which is mainly caused by the transfacial mechanical fixation devices [11, 14].

The major limitation of this technique is the wide incision necessary to complete the subcutaneous dissection, long operative time, long hospitalization, and increased postoperative surgical site complications, such as infections, seromas, skin necrosis, and hematomas.

For these reasons, since LeBlanc first described laparoscopic hernia repair in 1993, it has been widely used, and several techniques have been developed to benefit from the advantages of minimally invasive surgery. However, when compared with laparoscopic repairs, the Rives–Stoppa technique repair has the lowest recurrences and major complication rates [13].

In fact, the placement of an intraperitoneal mesh, often used in laparoscopic approaches, represents a risk for adhesions with the abdominal viscera, which can lead to lesions of the small bowel loops enteric fistulae, infections*,* and prosthesis displacement, despite the recent technological advances in the introduction of biocompatible prosthetic materials [12].

The most commonly performed surgical procedure to treat diastasis recti is abdominoplasty with plication of the anterior rectus sheath, where a wide laparotomy and an extended dissection of the subcutaneous tissue is required. This technique is associated with high risks of infections, seromas, and significant postoperative pain, with an unclear durability of the plication over time, as shown by several studies [15, 16].

The present technique is a variation of Costa’s technique described in Hernia [10]. Our approach combines the advantages of laparoscopic surgery, with the consolidated results of the Rives–Stoppa repair. It is based on the use of a linear stapler to join the posterior sheaths of rectus muscles to efficaciously repair the diastasis recti and a possible coexisting midline defect, as well as creating at the same time a space for the placement of a retromuscular mesh.

The reconstruction of the midline using a stapler is a great advantage because it is easy and fast, thereby reducing operating time, distributing the tension evenly on the suture, and preventing fascial tearing. In fact, it is crucial to have an equally distributed tension on the suture. Therefore, it is very important to carefully dissect the preperitoneal adipose tissue posteriorly to the midline, to fully expose the defect and to avoid any residue of adipose tissue between the jaws of the stapler.

An advantage of this technique is the concurrent closure of any midline defect with the stapler, thereby restoring the anatomy and minimizing the risk of recurrence. Chelala et al. in a paper published in Hernia in 2016 demonstrated the importance of closing the abdominal wall defect [17]. They conducted a study on 1326 patients who underwent laparoscopic hernia repair with routine suturing of the hernia gap and showed very good results in terms of recurrence and complications. However, laparoscopic sutures are technically difficult to perform. They require advanced skills and a long learning curve, contrary to the use of a stapler [8, 17].

The intraperitoneal approach allows to remove the preperitoneal adipose tissue, that otherwise might be included into the suture line, decreasing its strength; furthermore, this allows to discover the presence of epigastric hernias, that otherwise would be misdiagnosed. Also, the intraperitoneal technique allows to carefully monitor repetitive firing of the stapling device, avoiding damage to the intraperitoneal structures.

In case of diastasis recti below the arcuate line, our technique is not indicated, since the repair is based on the plication of the anterior fascia only. This can be performed by extending the sovrapubic port incision laterally (2–3 cm) and plicating the anterior fascia of recti muscles using a 2/0 barbed suture. Alternatively, THT technique can be used [12]. However, this technique has the following limits: (a) it does not allow to carry out the abdominoplasty since it would be necessary to detach the umbilicus from its base; (b) it does not allow to remove the preperitoneal adipose tissue, which would be included in the suture line weakening it.

An important advantage of our technique is the reduction of postoperative pain, mainly due to the absence of direct tacking of the mesh on the peritoneum, which is generally responsible for pain in laparoscopic repairs. As opposed to Costa’s article, we support not fixing the mesh because of its retromuscular placement in a perfectly shaped pocket created by the junction of the posterior sheaths of the recti abdominis. Owing to this particular positioning, the mesh is held in place by intra-abdominal pressure, according to the La Place principle [18].

To this date, we are not able to state whether the use of a reinforced stapler provides better results in terms of long-term recurrence. We used a reinforced recharge in 20 patients, but in early follow-ups, there were no differences between the two groups.

## Conclusions

This procedure was performed on a small number of patients and longer follow-up is needed. The short-term outcomes showed good results in terms of postoperative pain and decrease of preoperative symptoms. This technique is feasible and requires a short operating time. Associated ventral hernias can be repaired easily. Since this procedure avoids disconnection of the umbilicus, in contrast to other similar laparoscopic procedures, it allows synchronous abdominoplasty.

## Supplementary Information

Below is the link to the electronic supplementary material.

Supplementary file2 (MP4380831 KB)
